# Canines and carnassials as indicators of sociality in durophagous hyaenids: analyzing the past to understand the present

**DOI:** 10.7717/peerj.10541

**Published:** 2020-12-15

**Authors:** Juan Antonio Pérez-Claros, Carlos Coca-Ortega

**Affiliations:** Departamento de Ecología y Geología, Facultad de Ciencias, Universidad de Málaga, Málaga, Spain

**Keywords:** Hyaenidae, Evolution, Durophagy, Ecomorphology, Canines, Carnassials, Dentition, Carnivores

## Abstract

We analyzed the lower and upper dentition of the family Hyaenidae along its evolutionary history from a multivariate point of view. A total of 13,103 individual measurements of the lengths and widths of canines and the main post-canine teeth (lower third and fourth premolar, lower first molar, and upper second, third, and fourth premolars) were collected for 39 extinct and extant species of this family. We analyzed these measurements using principal component analyses. The multivariate structure characterized the main groups of previously defined hyaenid ecomorphs. Strikingly, our analyses also detected differences between social hunting durophages (such as *Crocuta crocuta*) and solitary scavengers (such as *Hyaena hyaena* or *Parahyaena brunnea*). Concerning the hyaenid bauplan, social hunters have large carnassials and smaller canines, whereas solitary scavengers show the exact opposite morphological adaptations. Additionally, scavengers exhibited upper canines larger than lower ones, whereas hunters have upper and lower canines of similar size. It is hypothesized that sociality has led to an increase in carnassial length for hunting durophages via scramble competition at feeding. Such competition also penalizes adults from bringing food to cubs, which are consequently breastfed. On the other hand, it is also hypothesized that natural selection has led to solitary scavengers having large canines to transport carcasses to cubs. Our results indicate that these functional aspects are also better reflected by lower teeth than the upper dentition, which leads to a mosaic evolution.

## Introduction

Canines serve crucial functions for carnivores as weapons for attack and defense. Nevertheless, they are also involved in many other general activities such as feeding (Van Valkenburgh, 1996), or more specific roles such as tearing off a tree bark in the case of ursids (Christiansen, 2008). Their comparatively simple morphology makes it very difficult on many occasions to know if canines have been adapted to specific secondary functions via natural selection or if they can accomplish some roles as mere exaptations (*sensu* Gould & Vrba, 1982).

In some cases, even without living representatives, it is possible to support an adaptive meaning for certain traits of canines, such as its hypertrophy in sabertooths (Antón, 2013). Nevertheless, functional interpretations usually require observations of living carnivores to link form and function. Some physical properties of canines, such as the ability to resist bending forces (Van Valkenburgh & Ruff, 1987; Christiansen & Adolfssen, 2005; Christiansen, 2007; Christiansen, 2008) or the force exerted by them (Christiansen & Adolfssen, 2005; Wroe, McHenry & Thomason, 2005; Christiansen & Wroe, 2007), have been analyzed in living species. Additionally, it is also important to place these morphologies in the context of their extinct relatives, as phylogenetic-historical aspects (Seilacher, 1970) can imprint morphological inertia without an adaptive meaning at present.

As opposed to canines, the carnassials of Carnivora (lower first molar and upper fourth premolars) show a morphology with comparatively higher complexity, which can be associated with different functions. The lower first molar typically has two functions. Its anterior part (the trigonid, whose two main cusps are the paraconid and the protoconid) acts as a blade, whereas its posterior half, the talonid, acts as a grinding basin (Van Valkenburgh, 1989; Van Valkenburgh, 1991). In some taxa, the metaconid (the third cusp of the trigonid) is developed, adding additional functional diversity to the lower first molar.

**Table 1:** Sample sizes for species and variables used in this study. Lc, Lp3, Lp4, and Lm1: lengths of the lower canine, third and fourth lower premolars, and the first lower molar, respectively. LC, LP2, LP3, and LP4: lengths of the upper canine, and second, third, and fourth upper premolars, respectively. Wc, Wp3, Wp4, and Wm1: widths of the lower canine, third and fourth lower premolars, and the first lower molar, respectively. WC, WP2, WP3, and WP4: widths of the upper canine, and second, third, and fourth upper premolars, respectively.

**Morphotype/Species**	**Abbrev.**	**Lc**	**Wc**	**Lp3**	**Wp3**	**Lp4**	**Wp4**	**Lm1**	**Wm1**	**LC**	**WC**	**LP2**	**WP2**	**LP3**	**WP3**	**LP4**	**WP4**
**Civet-like**																	
*Protictitherium crassum*	Pcra	11	11	60	59	65	64	75	73	10	8	14	14	16	15	18	19
*Protictitherium thessalonikensis*	Pthe									2	2	4	4	5	5	4	4
**Mongoose-like**																	
*Plioviverrops faventinus*	Pfav	2	2	2	2	3	3	4	3	1	1	3	3	2	2	3	3
*Plioviverrops guerini*	Pgue	1	1	5	4	4	3	4	4								
*Plioviverrops orbignyi*	Porb	7	7	13	13	12	13	15	15	7	7	14	14	15	14	16	15
**Jackal/Wolf-like**																	
*Hyaenictitherium hyaenoides*	Hhya	15	13	48	46	47	49	50	48	16	13	46	44	56	58	47	49
*Hyaenictitherium minimum*	Hmin	2	2	7	7	9	9	10	8	4	3	3	3	4	4	2	1
*Hyaenictitherium parvum*	Hpar	15	13	27	23	26	25	18	18	12	9	14	11	23	20	17	14
*Hyaenotherium wongii*	Hwon	12	11	134	133	127	126	117	120	10	10	115	123	144	151	151	142
*Ictitherium ebu*	Iebu									1	1	1	1	1	1	1	1
*Ictitherium ibericum*	Iibe	1	1	2	2	2	2	1	1	1	1	4	4	4	4	3	3
*Ictitherium pannonicum*	Ipan	2	3	11	7	9	7	8	7	1	1	1	1	2	2	3	3
*Ictitherium viverrinum*	Iviv	15	15	59	58	50	57	53	53	8	8	32	29	45	44	46	42
*Miohyaenotherium bessarabicum*	Mbes	3	3	3	3	5	3	3	3	4	3	4	2	4	4	6	5
*Thalassictis montadai*	Tmon	4	4	5	4	4	3	5	5	2	2	2	2	2	2	2	2
*Thalassictis robusta*	Trob	1	1	4	4	6	6	6	6	2	2	2	1	3	3	3	3
*Thalassictis spelaea*	Tspe	13	14	13	13	10	10	13	13	10	15	9	9	12	12	11	12
**Cursorial bone-meat eater**																	
*Chasmaporthetes australis*	Caus	2	2	6	6	7	7	6	6								
*Chasmaporthetes bonisi*	Cbon									1	1	2	2	2	2	2	2
*Chasmaporthetes gansgriensis*	Cgan	1	1	2	2	2	2	2	1								
*Chasmaporthetes lunensis*	Clun	18	19	27	25	32	32	32	34	12	12	25	25	36	36	40	36
*Chasmaporthetes ossifragus*	Coss	1	1	5	5	7	5	5	3								
*Hyaenictis aff. almerai*	Hafalm	2	2	2	2	2	2	2	2	1	1	2	2	2	2	2	2
*Hyaenictis almerai*	Halm	1	1	1	1	1	1	1	1								
*Hyaenictis hendeyi*	Hhen	1	1	3	3	5	5	3	3								
*Hyaenictis wehaietu*	Hweh	3	3	3	2	4	3	4	4	1	1	1	1	1	1	1	1
*Werdelinus africanus*	Wafr	3	2	4	3	3	4	2	2								
**Transitional bone-cracker**																	
*Ikelohyaena abronia*	Iabr	8	8	16	18	21	23	20	21	3	3	8	8	9	9	6	9
*Metahyaena confector*	Mcon	1	1	1	1	1	1		1								
*Palinhyaena reperta*	Prep	3	3	22	24	25	26	19	20	2	2	16	17	17	18	17	18
*Tongxinictis primordialis*	Tpri									1	1	2	2	2	2	1	1
**Fully developed bone cracker**																	
*Adcrocuta eximia*	Aexi	20	13	110	105	107	99	94	96	10	9	83	76	96	93	94	84
*Crocuta crocuta* (fossil)	Ccrof	152	135	284	223	303	244	263	228	114	116	123	100	188	150	166	138
*Crocuta crocuta* (living)	Ccrol	19	19	19	19	19	19	19	19	17	17	19	19	19	19	19	19
*Crocuta dietrichi*	Cdie	1	1	19	20	15	16	13	12								
*Hyaena hyaena* (fossil)	Hhyaf	15	15	21	22	24	22	20	22	13	13	22	21	30	32	29	27
*Hyena hyena* (living)	Hhyal	14	14	16	16	16	16	16	16	17	17	17	17	17	17	17	17
*Pachycrocuta brevirostris*	Pbre	33	28	108	90	108	92	90	83	24	22	45	38	58	48	55	49
*Parahyaena brunnea* (fossil)	Pbruf	3	3	11	11	8	7	9	7								
*Parahyena brunnea* (living)	Pbrul	15	15	15	15	15	15	15	15	15	15	15	15	15	15	15	15
*Parahyaena howelli*	Phol	5	5	7	7	5	7	4	5								
*Pliocrocuta perrieri*	Pper	30	26	103	94	115	104	103	91	21	22	45	37	59	47	66	51

Evolutionary context is essential to investigate the relationship between canines and carnassials in hyaenids because this family shows numerous autapomorphies. However, because there are only four highly derived species, it is necessary to study the rich fossil record of this family (Werdelin & Solounias, 1991; Turner, Antón & Werdelin, 2008). In the past, this family has not only shown high diversity, but also a rich disparity of ecological types (or ecomorphs). According to Werdelin & Solounias (1996), hyaenid species can be assigned to six ecological categories: (1) civet-like insectivores/omnivores, (2) mongoose-like insectivores/omnivores, (3) jackal- and wolf-like meat and bone eaters, (4) cursorial meat and bone eaters, (5) transitional bone crackers, and (6) fully developed bone crackers (*Proteles cristatus* could be considered as the only member of the seventh category: specialized termite eater). In this respect, Coca-Ortega & Pérez-Claros (2019) analyzed the multivariate pattern of the main elements of the lower and upper post-canine dentition for this family along its evolutionary history utilizing principal component analyses. The two first components characterized the main groups of ecomorphs, whereas the species of scavenging and hunting durophagous hyaenids were differentiated into two well-defined clusters along the third axis. The three living species of bone crackers were essential to the interpretation of the ecomorphological meaning of the axis because they were allocated in each of these two groups according to their ecological niche.

**Table 2:** Mean values in mm for the lengths and widths of the upper and lower canines for the species of hyaenids analyzed. The sample sizes are in brackets.

**Morphotype/Species**	**Lc Mean (N)**	**Wc Mean (N)**	**LC Mean (N)**	**WC Mean (N)**
**Civet-like**				
*Protictitherium crassum*	6.5 (11)	4.7 (11)	7.7 (10)	5.3 (8)
*Protictitherium thessalonikensis*			5.9 (2)	4 (2)
**Mongoose-like**				
*Plioviverrops faventinus*	6.1 (2)	4.8 (2)	6.5 (1)	5.1 (1)
*Plioviverrops guerini*	5.2 (1)	4.7 (1)		
*Plioviverrops orbignyi*	4.6 (7)	3.8 (7)	4.6 (7)	3.3 (7)
**Jackal/Wolf-like**				
*Hyaenictitherium hyaenoides*	11.9 (15)	8 (13)	12.4 (16)	8.6 (13)
*Hyaenictitherium minimum*	8.8 (2)	7.6 (2)	8.9 (4)	6.6 (3)
*Hyaenictitherium parvum*	12.8 (15)	8.7 (13)	12.6 (12)	9.3 (9)
*Hyaenotherium wongii*	11 (12)	7.7 (11)	11.4 (10)	8.1 (10)
*Ictitherium ebu*			10.7 (1)	6.7 (1)
*Ictitherium ibericum*	9.5 (1)	7 (1)	11.5 (1)	7.5 (1)
*Ictitherium pannonicum*	12.1 (2)	7.8 (3)	13.6 (1)	8.9 (1)
*Ictitherium viverrinum*	9.7 (15)	7.3 (15)	10.8 (8)	7 (8)
*Miohyaenotherium bessarabicum*	12.3 (3)	9.3 (3)	13.2 (4)	8.1 (3)
*Thalassictis montadai*	13.6 (4)	10.5 (4)	14.3 (2)	10.5 (2)
*Thalassictis robusta*	10.2 (1)	6.9 (1)	8.7 (2)	6 (2)
*Thalassictis spelaea*	9.1 (13)	6 (14)	9.3 (10)	6.3 (15)
**Cursorial bone-meat eater**				
*Chasmaporthetes australis*	17.3 (2)	12.5 (2)		
*Chasmaporthetes bonisi*			15 (1)	12 (1)
*Chasmaporthetes gansgriensis*	16.2 (1)	11.5 (1)		
*Chasmaporthetes lunensis*	16.5 (18)	12.1 (19)	17.5 (12)	13 (12)
*Chasmaporthetes ossifragus*	17 (1)	13.2 (1)		
*Hyaenictis aff. almerai*	18.7 (2)	13.2 (2)	18.8 (1)	13.2 (1)
*Hyaenictis almerai*	14 (1)	9 (1)		
*Hyaenictis hendeyi*	15.5 (1)	11 (1)		
*Hyaenictis wehaietu*	13.5 (3)	10.4 (3)	14.2 (1)	10.1 (1)
*Werdelinus africanus*	16 (3)	12.5 (2)		
**Transitional bone-cracker**				
*Ikelohyaena abronia*	12.7 (8)	9.6 (8)	11.9 (3)	9.2 (3)
*Metahyaena confector*	9.8 (1)	7.2 (1)		
*Palinhyaena reperta*	10.7 (3)	7.6 (3)	12.1 (2)	7.5 (2)
*Tongxinictis primordialis*			11.7 (1)	8 (1)
**Fully developed bone cracker**				
*Adcrocuta eximia*	16.6 (20)	13.3 (13)	17.2 (10)	13 (9)
*Crocuta crocuta* (fossil)	16.4 (152)	13.6 (135)	16.8 (114)	13.3 (116)
*Crocuta crocuta* (living)	14.9 (19)	11.5 (19)	16 (17)	11 (17)
*Crocuta dietrichi*	12.8 (1)	9.6 (1)		
*Hyaena hyaena* (fossil)	12.9 (15)	9.8 (15)	13.3 (13)	9.2 (13)
*Hyena hyena* (living)	14.3 (14)	10.6 (14)	14.1 (17)	10 (17)
*Pachycrocuta brevirostris*	21.5 (33)	16.9 (28)	20.8 (24)	15.2 (22)
*Parahyaena brunnea* (fossil)	16.8 (3)	12.3 (3)		
*Parahyena brunnea* (living)	16.9 (15)	12.8 (15)	16.1 (15)	11.8 (15)
*Parahyaena howelli*	13.6 (5)	10.9 (5)		
*Pliocrocuta perrieri*	18.1 (30)	13.6 (26)	17.9 (21)	13.2 (22)

Canine teeth were excluded from these analyses because they are unknown for many extinct species. Their simple root condition makes it easier for them to detach from the alveoli and become lost during the fossilization process. Van Valkenburgh & Ruff (1987) hypothesized that the relatively round canines of living hyenas could be an adaptation to scavenging, simply reflect ancestry, or that they might serve some other functions. In the present study, canines were incorporated into the analyses with a double objective. On the one hand, our study intended to contrast whether after incorporating the canines, the structure of the covariance between the different variables of the dentition changed or was maintained with respect to that obtained by Coca-Ortega & Pérez-Claros (2019) using only the post-canine dentition. The second goal was to deepen the functional meaning of the canines by correlating them with the rest of the dentition.

## Material & Methods

The analyzed sample of hyaenids comprised 36 extinct species and three living durophagous species, whose fossil representatives have been considered as different observations. This is a subset of the accepted species of hyaenids (approximately 70 species) whose canines are known. Nevertheless, it covers the entire ecomorphological spectrum of this family according to the basic types defined by Werdelin & Solounias (1996) and summarized in Turner, Antón & Werdelin (2008). The taxonomy used follows that of Werdelin & Solounias (1991) and Turner, Antón & Werdelin (2008). Six new species of previously accepted genera and one belonging to a new genus (*Werdelinus africanus*) described later, have been assumed to be valid. Although some measurements were taken from museum specimens, most of the data came from 132 bibliographic sources written in 11 different languages: English, French, German, Spanish, Russian (Cyrillic), Chinese (Hanzi), Dutch, Greek (in the Greek alphabet), Catalan, Basque, and Italian. All details on data provenance are provided in the study by Coca-Ortega & Pérez-Claros (2019).

The database comprised 13,103 individual measurements of anteroposterior lengths (L) and buccolingual widths (W) for the lower (c, p3, p4, and m1) and the upper (C, P2, P3, and P4) dentition (Table 1). All the data for the post-canine dentition were obtained from the study of Coca-Ortega & Pérez-Claros (2019), whereas the measurements for the canines are shown in Table 2 and Table S1.

Principal component analyses (PCA) were performed for the means of the lower and the upper dentition using variance–covariance matrices because all the variables were in the same units. Given that either the upper or the lower dentition was unknown for some of the species, the number of species for each analysis differed (31 and 38, respectively).

Between-groups principal component analyses (bgPCA) were computed using PAST v. 3.24 (Hammer, Harper & Ryan, 2001). Unlike PCA, in the case of bgPCA, eigenanalyses are conducted using the means of previously defined groups. Consequently, eigenvalues and eigenvectors were obtained from the variance–covariance matrices between the ecomorph means. This allowed for the counterbalance of the comparatively low number of cases for civet-like and mongoose-like hyaenids because each ecomorph had the same weight in the analyses regardless of the number of species contained in it. Because the importance of a given principal component may not always be reflected by the size of its associated eigenvalue (Jolliffe, 1982; Jolliffe, 2002, p. 174), the criterion adopted here (as in Coca-Ortega & Pérez-Claros, 2019) was to retain the three first components, because they showed a clear biological meaning.

A sample of dental measurements for living canids and felids (29 and 21 species, respectively) was also obtained from bibliographic sources for comparative purposes (Table S2).

## Results

### Multivariate analyses

The multivariate pattern for both datasets, the upper and lower dentition, was quite similar to the results obtained by Coca-Ortega & Pérez-Claros (2019) for the post-canine dentition (although including the canines appreciably reduced the sample size). Consequently, this similarity in covariance structure also provided us an approximate estimate regarding the position in morpho-space for those species whose canines were not preserved.

The first principal components accounted for more than 99% of the variance for both analyses (Table 3). Given that all the loadings were positive in both analyses, these components were size axes. Size was the most important source of variation in our data because the sample was composed of dentitions for animals ranging from the size of a mongoose to that of a small lion.

**Table 3:** Between-groups principal component loadings and percentage of variance explained for the two analyses.

	**Variable**	**PC I**	**PC II**	**PC III**
**Lower dentition**	Lc	0.354	−0.076	0.501
	Wc	0.259	0.265	0.629
	Lp3	0.404	−0.248	−0.148
	Wp3	0.302	0.718	−0.154
	Lp4	0.453	−0.440	0.156
	Wp4	0.274	0.359	−0.135
	Lm1	0.474	−0.146	−0.489
	Wm1	0.220	0.053	−0.161
	Eigenvalue	161.87	0.93	0.31
	% variance	99.23	0.57	0.19
**Upper dentition**	LC	0.294	−0.082	0.511
	WC	0.217	0.074	0.704
	LP2	0.289	−0.444	0.119
	WP2	0.208	0.420	0.082
	LP3	0.392	−0.106	−0.023
	WP3	0.279	0.706	−0.136
	LP4	0.630	−0.266	−0.443
	WP4	0.334	0.184	−0.086
	Eigenvalue	245.88	1.11	0.53
	% variance	99.21	0.45	0.21

**Figure 1:**
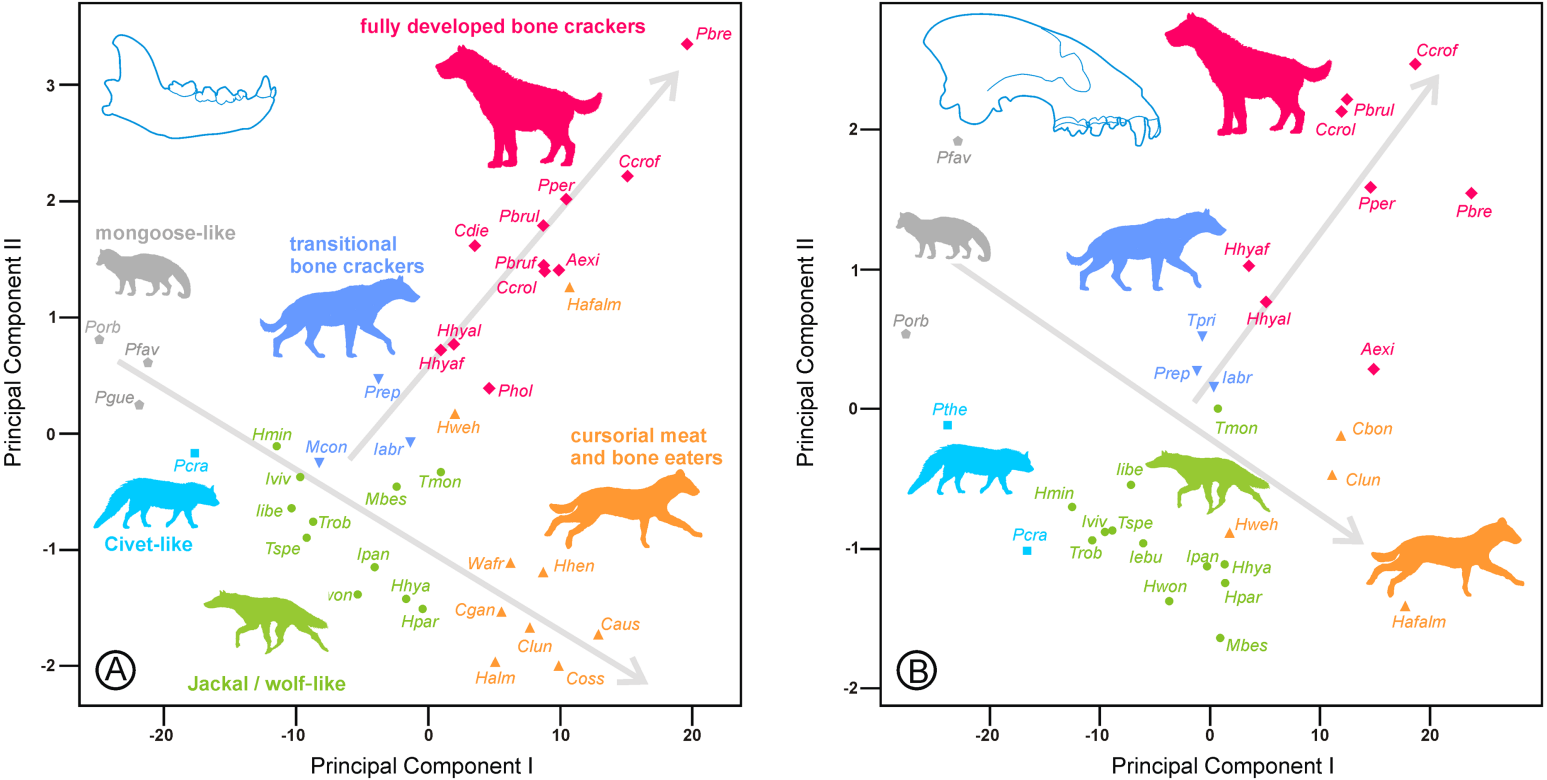
Bivariate plots of the scores on the two first between-groups principal components (A) for the lower dentition and (B) for the upper dentition. Gray lines indicate morphological trends. Abbreviations as in Table 1.

The second principal components only explained approximately 0.5% of the variance in both cases; however, they were very informative from a biological point of view. All the loadings for lengths were negative, whereas those for widths were positive, particularly for the third premolars (Table 3). Consequently, the second components were shape axes, where dentitions were arranged from long and narrow shearing teeth to wide and stout crushing teeth. The plots of species scores for components I and II (Fig. 1) show a specific allocation for six ecomorphotypes consistent with the lower and upper dentitions. The arrangement of these ecomorphs defined two clear morphological trends from the jackal/wolf-like hyaenids, one of them toward the cursorial meat and bone eaters and the other to the fully developed bone crackers.

All species had approximately the same relative position in both plots, except *Hyaenictis* aff. *almerai*, which showed a dentition in mosaic because its lower teeth were typical of bone-cracking hyaenids, whereas its upper dentition was distinctive of meat and bone eaters.

**Figure 2:**
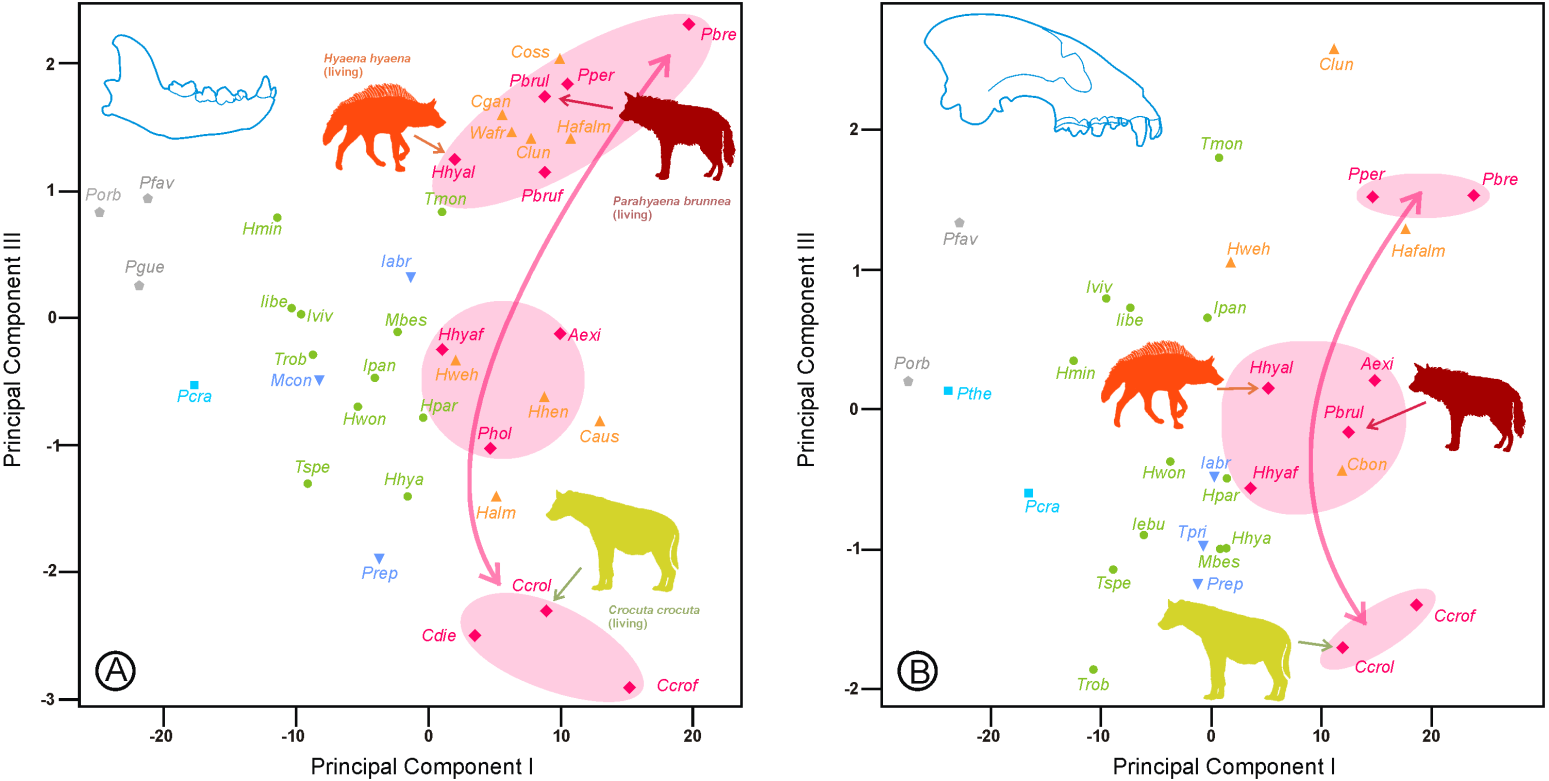
Bivariate plots of the scores on the first and third between-groups principal components (A) for the lower dentition and (B) for the upper dentition. Shaded areas correspond to groups of durophagous species discussed in the text, as well as the line connecting them that represents a gradient from scavenging to hunting durophagous adaptations. Colors and symbols as in Fig. 1. Abbreviations as in Table 1.

The most important variables in the third principal components were the lengths of the carnassials and the size of the canines (Table 3). It is worth noting that the variance in the canines not attributable to size surprisingly correlated with this third axis, which separated hunting from scavenging species within the durophagous ecomorphotype (Fig. 2).

The species of durophagous hyenas appear to be distributed into three groups along a scavenging-hunting gradient for both analyses, although there are certain differences in the species that constitute such groups. In the case of the lower dentition, most species were grouped with those inferred from the post-canine dentition according to Coca-Ortega & Pérez-Claros (2019), except *Adcrocuta eximia* and the fossil representatives of *Hyaena hyaena*, which were in an intermediate position (*Parahyaena howelli* exhibits an intermediate morphology for the post-canine dentition). In the case of the third principal component of the upper dentition, the living representatives of *H. hyaena* and *P. brunnea* were incorporated into the group with intermediate morphologies.

Another important difference between both analyses was that durophagous species (either scavenging or hunting) were at both ends of the third principal component for the lower dentition (Fig. 2A) but not for the upper dentition (Fig. 2B), which implies that the morphological variation for the rest of the ecomorphs (mainly within the jackal/wolf ecotype) was also important in this component.

Regarding the lower dentition, scores on the third principal component showed a clear sequence of increasing specialization along the lineage of *Parahyena*: *P. howelli, P. brunnea* (fossil), and *P. brunnea* (living). Similar results were obtained for fossil and living *H. hyaena*.

### Bivariate analyses of canines and carnassials

Because only three variables were involved in the third principal component, it was viable to join the length and width of canines (which were positively correlated) into a new variable defined as the square root of their product. This allowed us to examine the behavior of carnassials and canines using bivariate plots and to incorporate the living families of top predators (canids and felids) for comparative purposes.

**Figure 3:**
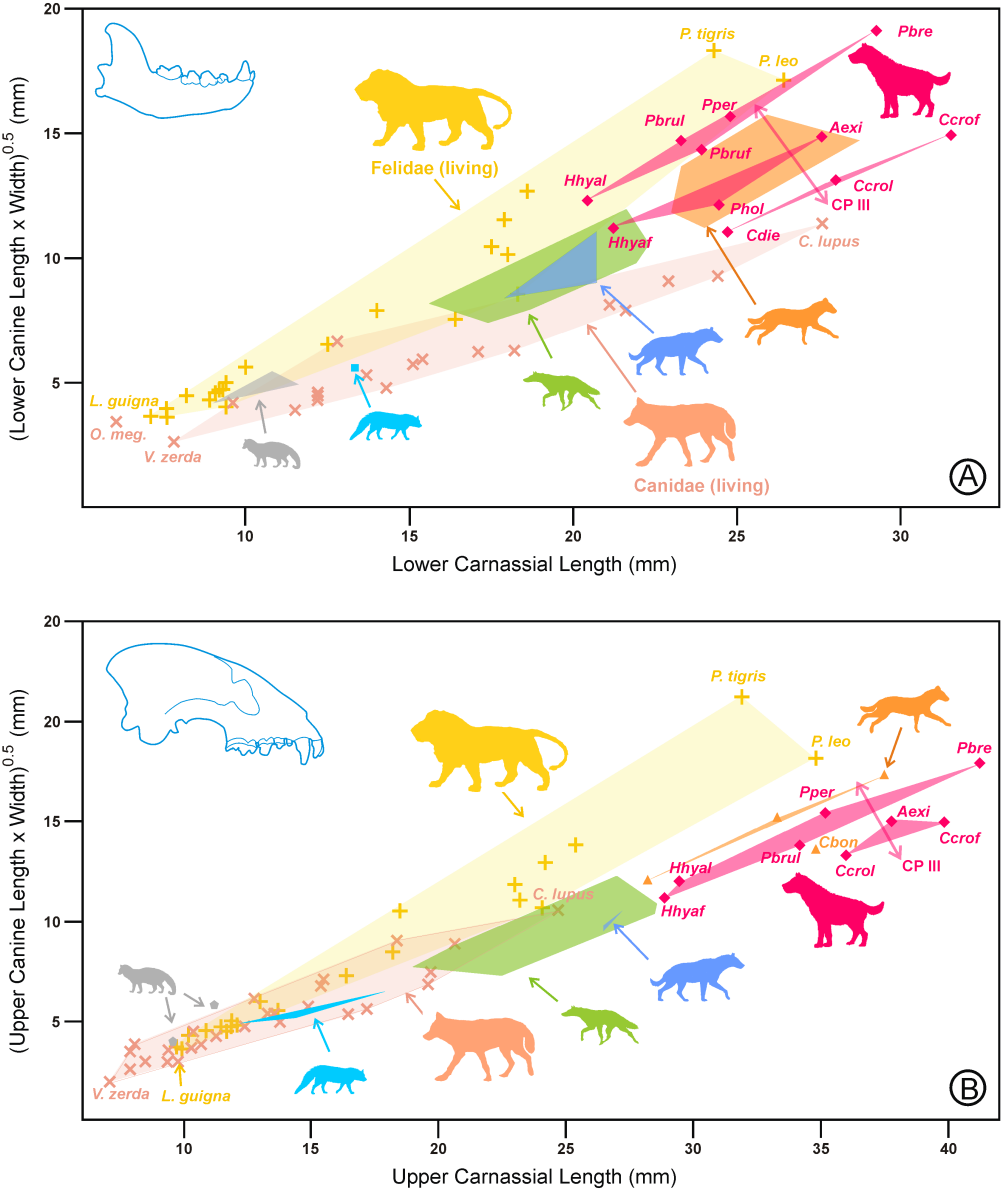
Bivariate plots of the canine size on the carnassial length for (A) the lower dentition and (B) the upper dentition in hyaenids and living representatives of canids and felids. Symbols, colors, and abbreviations for hyaenids as in Fig. 1. Shaded areas correspond to convex hulls for the two living families and the hyaenid ecomorphs. Red lines represent the variation of the durophagous hyaenids along with the third principal component. All data are in Table S1.

As shown in Fig. 3, for a given carnassial length, the upper and lower canines of scavenging durophages were larger than those of hunting ones. The plot of the canine size on the carnassial length for the lower dentition (Fig. 3A) shows that hyaenids are located approximately between felids and canids. The durophagous ecotype was distributed into three sets that corresponded to the groups in Fig. 2. The intermediate group was situated at the center of the cursorial ecomorph, following approximately the general rule for the family Hyaenidae. However, more advanced scavenging durophages overlapped with felids, whereas hunting durophages were relatively close to canids. In relation to the upper dentition (Fig. 3B), there were some differences, because hyaenids (excluding hunting bone crackers) approximately follow the morphological trend shown by canids. Another difference is that, although the relative position of the durophages resembles that shown in Fig. 2B, the species appear to be grouped into two (rather than three) sets. In this case, scavenging durophages appeared to continue the trend that followed the rest of the ecomorphs, whereas hunting durophagous species show a reduction in the upper canine with respect to the fourth upper premolar. In conclusion, the relationship between the carnassial length and canine size clearly shows the existence of two types of durophages.

### Relationships with body mass

At this point, it is important to clarify whether carnassials and/or canines have been reduced (or enlarged) in coordination with body mass. To this end, both variables have been plotted on body mass for a subset of durophagous species for which there were estimations of body masses. This has been carried out using published figures of body masses based on non-dental variables such as skull length or postcranial variables, to avoid circularity (see Table S2).

**Figure 4:**
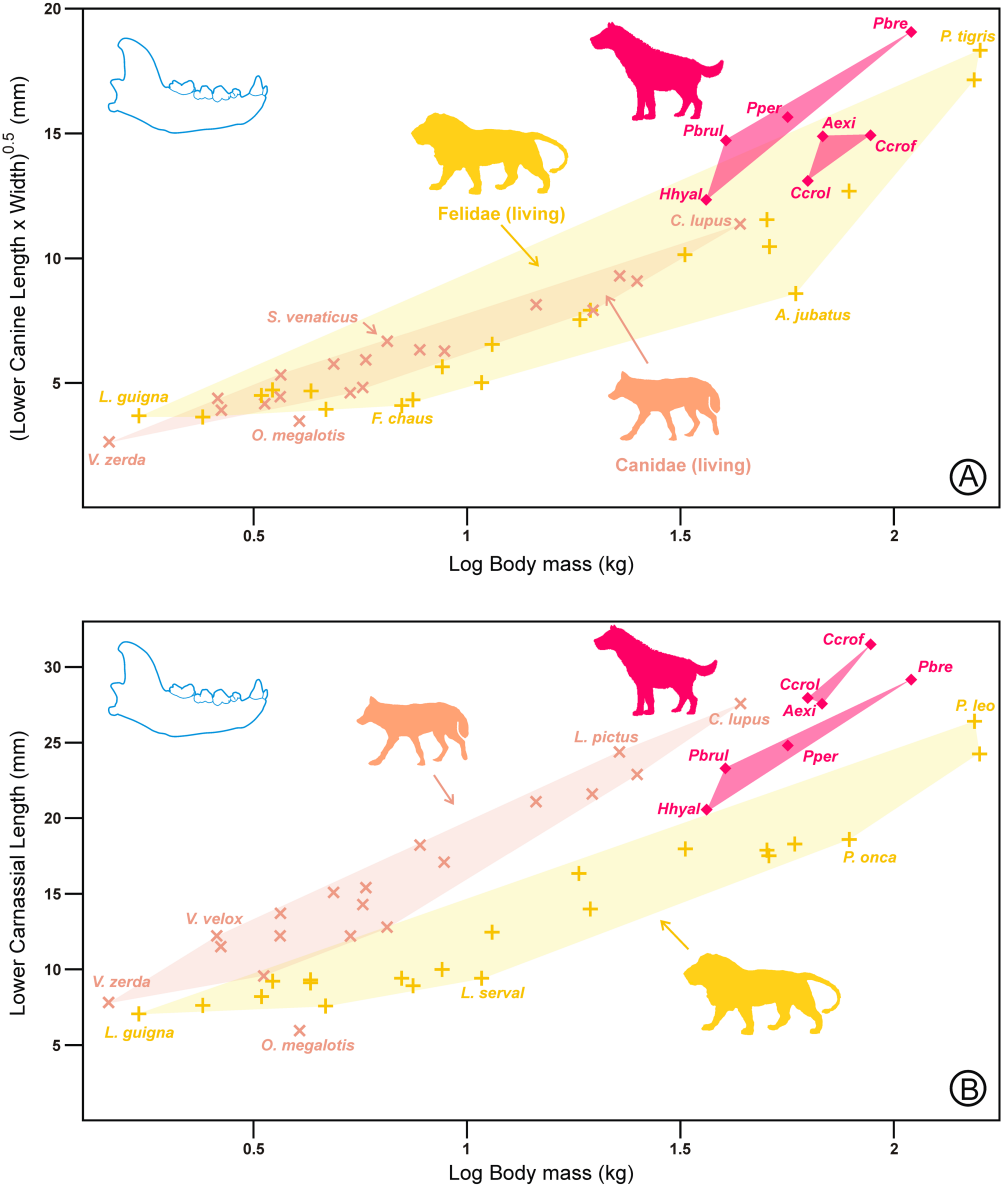
Bivariate plots of the canine size (A) and carnassial length (B) on body mass for the lower dentition. Symbols, colors, and abbreviations as in Fig. 3. Note in Fig. 4B that hunting durophages appear to continue the trend shown by canids but that impression can be deceptive from a functional point of view because m1 length in canids includes a large talonid that is practically absent in felids and very reduced in these hyaenids. All data are in Table S2.

Figure 4A shows that for a given body mass, hyenas have larger canines than canids and felids, being larger in the scavenging durophages than in the hunting ones. On the contrary, Fig. 4B shows the opposite trend for carnassial length. In the latter case, hyaenids are intermediate between canids and felids.

**Figure 5:**
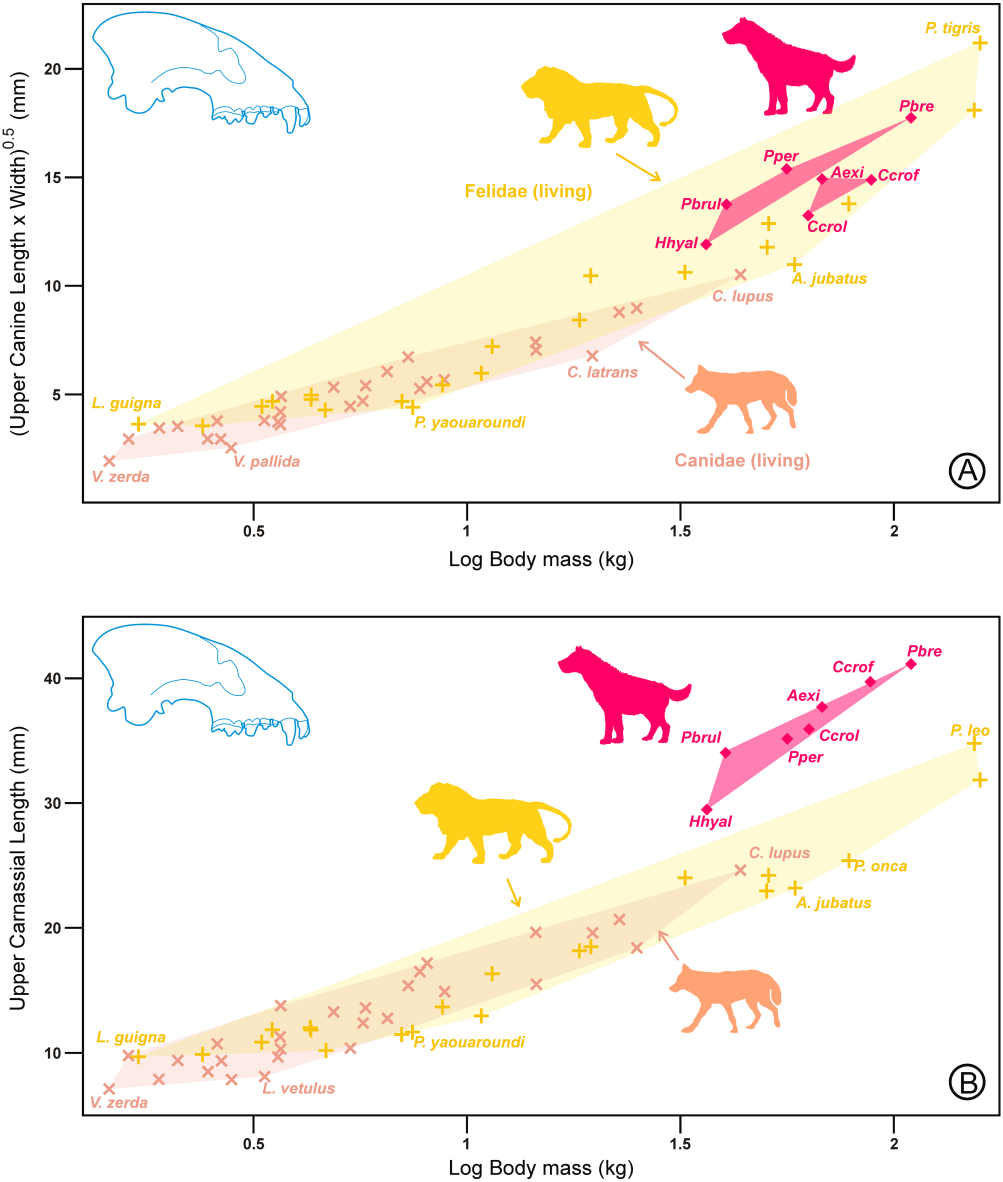
Bivariate plots of the canine size (A) and carnassial length (B) on body mass for the upper dentition. Symbols, colors, and abbreviations as in Fig. 3. All data are in Table S2.

The results for the upper canine (Fig. 5A) were analogous to those of the lower canines, although the differences with respect to canids and felids were not as extreme. Again, hunting durophagous species appeared to follow the canid trend, whereas the scavengers were closer to felids. Finally, in the case of the upper carnassial length (Fig. 5B) for a given body mass, the durophages exhibited longer carnassials than any canid or felid. In this case, all the durophages projected on the same region of the morphospace (regardless of whether they were scavengers or hunters). Consequently, there was no differentiating adaptation for the upper carnassial length within the durophagous ecotype.

## Discussion

The results obtained here show that not only carnassials, but also canines are involved in the adaptations within the durophagous ecotype toward either a hunting or a scavenging ecology. A way to link the differences in morphology to specific functions is through the study of the ecology of living durophagous hyenas because they can be assigned to the two kinds of adaptations.

### Carnassial length

Increased slicing function for carnassials of hunting durophages, such as *C. Crocuta*, can be considered an indication of sociality as a consequence of the selective pressures involved in communal feeding. This species is organized into clans, which forage, hunt, feed, and defend territories (Kruuk, 1972; Mills, 1990; Henschel & Skinner, 1991; Holekamp et al., 1997; Cooper, Holekamp & Smale, 1999; Boydston, Morelli & Holekamp, 2001; Smith et al., 2008). Spotted hyena (*C. crocuta*) clans usually crowd around carcasses and devour them immediately (Kruuk, 1972; Mills, 1990). As described by Kruuk (1972, p. 124), carcasses became “completely buried beneath a writhing mass of hyaena bodies”. Although there is a clear dominance hierarchy within clans, all members can feed together and competition between feeding hyenas lies more in the speed of eating rather than in actual fighting (Mills, 1990). Consequently, the ability of an individual to feed rapidly not only determines the quality and quantity of food ingested by competing hyenas at fresh ungulate carcasses (Mills, 1990; Holekamp et al., 1997), but it is also essential for low-ranking hyenas if they individually capture prey. In fact, according to Holekamp et al. (1997), in those circumstances, a low-ranking hyena often has 1–15 min of competition-free time to feed before other hyenas arrive. During that time, an adult hyena can ingest 2–20 kg of flesh, which is the food that would equal or exceed its daily requirements. Consequently, the selective pressure to increase the speed of feeding can lead to an increased slicing function of carnassials in *C. crocuta*.

Conversely, *H. hyaena* and *P. brunnea* are essentially solitary scavengers that feed on the carrion of medium-sized mammals, although a wide variety of resources have also been systematically found in their scats. Striped hyena feces usually contain plant material (grass, fruit, pods, leaves, seeds, and grains), birds, insects, and a variety of small mammals such as rodents, hares, and other small carnivores such as civets or mongooses. These material as a whole can account for more than 50% of the percentage of occurrence in scats (Leakey et al., 1999; Mondal, Sankar & Qureshi, 2012; Alam & Khan, 2015). Kruuk (1976) reported that striped hyenas can cause considerable damage to diverse crops (e.g., melons, watermelons, cucumbers, peaches, dates, and grapes). Brown hyenas feed on the carrion of medium- to large-sized mammals; however, their scats also contain significant percentages of fruits (e.g., melons of *Citrullus* sp.), insects, small mammals (such as rodents or hares), and birds (Mills & Mills, 1978; Owens & Owens, 1978; Maude & Mills, 2005; Faure et al., 2019). From scat analyses, it is not possible to distinguish between the food items that had been killed and which had been scavenged, being necessary direct observations (Mills, 1992). Predation events for these two scavenging hyenas have been very rarely observed, being in all occasions on small mammals (e.g., hares, springhares, bat-eared foxes, small rodents) and by using an extremely unsophisticated technique (Kruuk, 1976; Owens & Owens, 1978; Mills, 1990). Hunting in *H. hyaena* and *P. brunnea* also appear to be rather ineffective, given that only between 4.7% and 13.7% of the attempts were successful (Owens & Owens, 1978; Mills, 1990). Consequently, there is a general agreement that hunting represents an insignificant contribution to the diet of these two species (Kruuk, 1976; Owens & Owens, 1978; Skinner & Ilani, 1979; Mills, 1990).

Another important difference between the living hunting and scavenging durophagous species is the number of animals feeding at the same time on the carcasses. As solitary foragers, both striped and brown hyenas consume most of the food alone. However, sometimes several brown hyenas will accumulate at large carcasses if there is sufficient food. In these circumstances, few hyenas (normally only one of them) feed at a time, whereas others wait for a chance (Owens & Owens, 1978; Mills, 1990). In the case of striped hyenas, some of them can also visit the same carcass over a long period but never simultaneously (Wagner, Frank & Creel, 2008). The situations where several striped hyenas have been seen feeding at the same time correspond to small groups of related animals visiting feeding sites established by humans in nature reserves (Macdonald, 1978; Skinner & Ilani, 1979). In conclusion, the ability to ingest food rapidly for striped and brown hyenas is not as crucial as in spotted hyenas.

Ewer (1954) presented an exhaustive study of the comparative functional morphology of carnassials and premolars for *C. crocuta* vs. *H. hyaena* and *P. brunnea*, consistent with the ecological observations presented above. This author correctly identifies these species not as part of a single trend, in which each of the three species under consideration has progressed to different extents, but as a part of two different trends towards different niches (*P. brunnea* being more advanced than *H. hyaena* in the corresponding trend). In her pioneering study, Ewer (1954) also highlighted that carnassials in *C. crocuta* are single-purpose structures adapted to slicing, whereas in *H. hyena* and *P. brunnea*, carnassials have two or even three functions: slicing, crushing, and chopping because the talonid of m1 occludes with M1. The results presented here for the living and fossil representatives of durophagous species are in accordance with the results obtained by Ewer (1954).

In this respect, the lack of differences in the upper carnassial length between the representatives of the two kinds of adaptations (Fig. 5B) is explained because the differences are not in the relative length of P4, but in the morphology of the cusps (and their associated functions). Therefore, in the case of scavengers, the protocone and the parastyle of P4 are involved in crushing, whereas in hunters such as *C. crocuta,* the upper fourth premolar is exclusively used for slicing (Ewer, 1954).

Another interesting issue is that adaptations in upper dentitions are delayed with respect to the lower dentition for both *P. brunnea* and *H. hyaena* (Fig. 2), which indicated a mosaic evolution, probably because the maxillary dentition is embedded in the cranium, which is a tradeoff between different functional demands (e.g., feeding, vision, and smell), whereas the mandible is only involved in food acquisition and processing (Figueirido et al., 2011).

In summary, the increase in the relative length of carnassials in the case of hunting durophagous hyaenids can be reasonably explained by the action of natural selection towards increasing the cutting component of the dentition, given the rapid need to cut skin, tendons, and other soft tissues. However, the differences in the size of canines between scavenging and hunting durophages are not so obvious.

### Canine size

Canines of durophagous hyaenids are morphologically more similar to those of felids than those of canids (Van Valkenburgh & Ruff, 1987). However, the hunting style of *C. crocuta* is similar to that shown by social hunters of the family Canidae (Kruuk, 1972; Mills, 1990; Holekamp et al., 1997); while on the other hand, *P. brunnea* and *H. hyaena* are not specialized hunters.

In the case of spotted hyenas, medium- to large-sized prey are grabbed and disemboweled by the belly, rump flank, or eviscerated in the anal region in the same manner as African wild dogs or dholes, which can perform these tasks without the large canines exhibited by felids. Additionally, *C. crocuta* can use their powerful premolars to laterally bite the flanks of their prey producing similar results. In any case, canines in hyaenids are larger than in any canid or felid for a given body mass (Figs. 4A and 5B). Consequently, it does not seem necessary to look for additional explanations for the relatively small size of canines for hunting durophages (with respect to the scavengers) because they are adequate to produce the slashing wounds associated with their style of hunting. Nevertheless, the reason for the increased canine size of scavenging hyenas cannot be considered a convergence with felids for hunting larger prey than themselves.

Although hyenas (as other carnivores) only use canines occasionally to consume bone (Van Valkenburgh, 1996), the relative increase in canine size seen in scavenging hyenas might be considered an adaptation to a harder diet. Such a diet would require greater bite efforts for fracturing bones (Figueirido, Tseng & Martín-Serra, 2013), which would increase the risk of canine breakage. According to Van Valkenburgh (2009), canine teeth for carnivores have a much higher frequency of fracture than any other teeth. However, the diet appears to be harder for spotted hyenas than for striped or brown hyenas. The percentage of individuals with at least one fractured tooth in the case of *C. crocuta* (57%) was significantly higher than that in *H. hyaena* (35%, *p* = 0.002) and *P. brunnea* (41%, *p* = 0.039). On the other hand, there were no significant differences in broken teeth between *P. brunnea* and *H. hyaena* (*p* = 0.276) (data from Van Valkenburgh, 2009). Spotted hyenas subject their teeth to greater efforts as deduced by the larger number of unidentified bone chips found in their dens compared with the dens of striped and brown hyenas (Skinner, 2006). On the other hand, the percentages of fractured canines reported by Van Valkenburgh (2009) for the three species are practically identical, with no statistically significant differences (9.6%, 8.9%, and 8.3% for *C. crocuta*, *P. brunnea*, and *H. hyaena*, respectively). Consequently, the harder diet hypothesis does not appear to hold.

However, there is another aspect that could help explain the relative increase in the size of the canine for the living scavengers *H. hyaena* and *P. brunnea* with respect to *C. crocuta*: the transport of carcasses to their cubs. Differences in denning behavior between living species are notable. Thus, while striped and brown hyenas feed their cubs by suckling and providing meat and bones, spotted hyenas raise their young basically by breastfeeding (Kruuk, 1972; Kruuk, 1976; Mills, 1983; Mills, 1984; Mills, 1990; Skinner, Henschel & Van Jaarsveld, 1986; Holekamp et al., 1997; Skinner, 2006; Lansing et al., 2009). *C. crocuta* is rarely observed transporting bones to their cubs (Lansing et al., 2009), and consequently, the number of bones in their dens rarely exceeds one hundred. On the contrary, the bone number is in the thousands in the case of dens of striped and brown hyenas (Skinner, Henschel & Van Jaarsveld, 1986; Skinner, 2006; Pokines & Kerbis-Peterhans, 2007; Lansing et al., 2009). The rates of bone accumulation in dens observed in long-term studies show figures of 817 bones per year in the case of brown hyenas (Skinner et al., 1998), in contrast to the 30.4 found for spotted hyenas (Pokines & Kerbis-Peterhans, 2007). Moreover, not only are the rates higher, but the transport distances are also longer. In the case of brown hyenas, bones are normally transported over 11–14 km to dens (Kuhn, Wiesel & Skinner, 2008), whereas approximately 90% of the bones brought to dens by spotted hyenas come from distances less than 1 km (Skinner, Henschel & Van Jaarsveld, 1986).

As an illustrative example, there was an observation of transport of pig remains to a striped hyena den placed at the North of the Negev Desert in Israel (Kerbis-Peterhans & Horwitz, 1992). Given the prohibition on eating pork for religious reasons, the remains of pigs could not have come from human settlements located in the area. The only possible source of pigs was located at 35 and 40 km from the den. Additionally, the majority of the bone elements accumulated by *H. hyaena* in some of their cubs belonged to species of considerable sizes, such as camels or horses (Kuhn, 2005), which can be heavy and difficult to transport.

An interesting point to consider is that, because the assemblages collected by brown hyenas are a reasonably good reflection of the composition of the adjacent fauna (Skinner & Van Aarde, 1991; Skinner et al., 1998; Skinner, 2006), the remains must certainly be transported from distant locations, given the low animal density in the arid ecosystems where they live. Brown hyenas travel mean distances of 30 km per night (Mills, 1990) and striped hyenas travel mean distances of 19 km per night (Kruuk, 1976), which allows the prospect of large areas.

**Figure 6:**
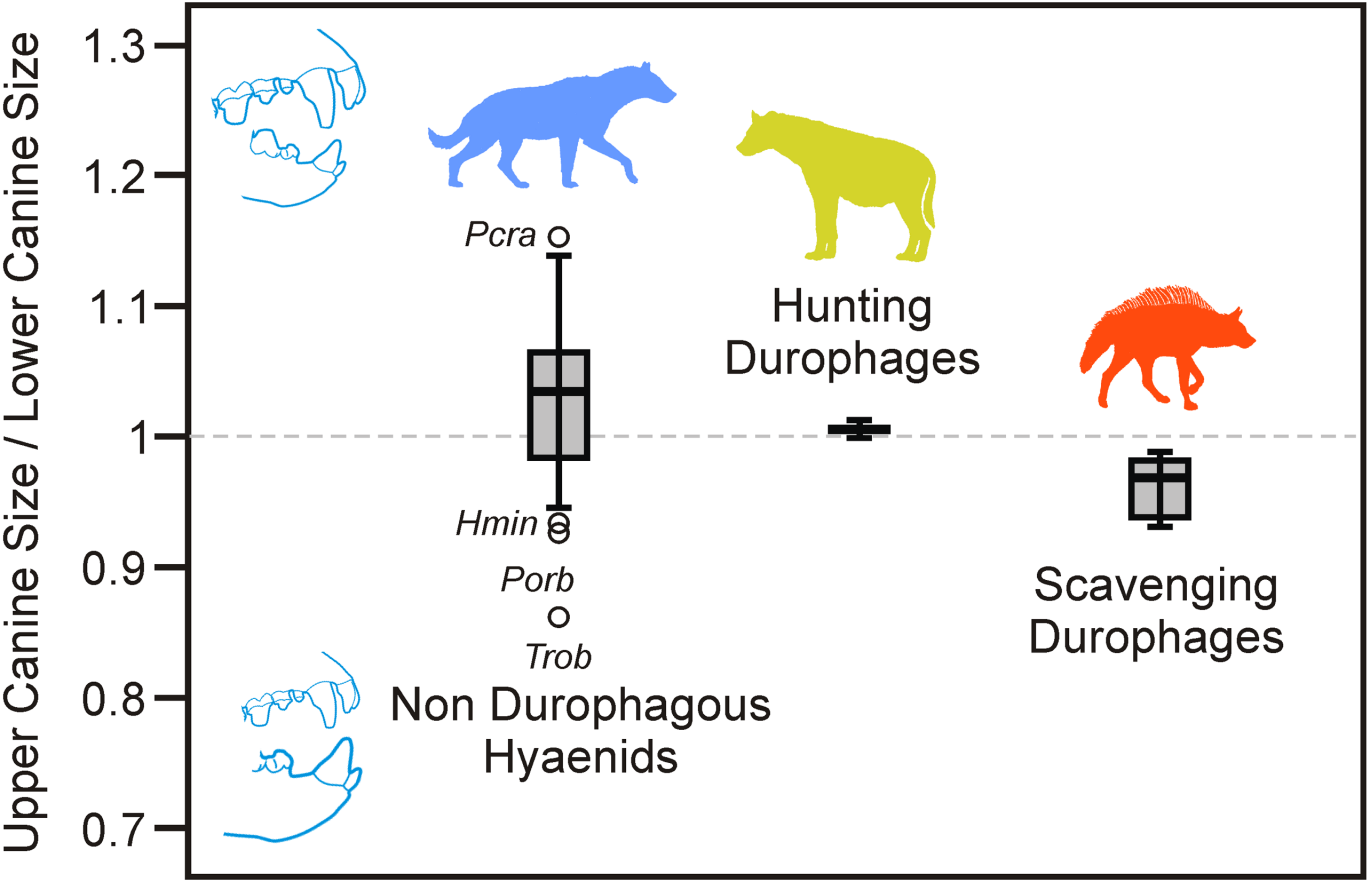
Box plot of the ratio between the upper canine and lower size. Note that the ratio is less than 1 for all the scavenging durophagous species, whereas it is virtually 1 for hunting durophages. Abbreviations for species as in Table 1. Data from Table 2.

A hypothetical expectation of the transport hypothesis (pending a more detailed biomechanical study) would be that the lower canines should be larger than the upper canines in transporting scavengers compared with non-transporting hunters. This hypothesis is based on the fact that the lower canines would bear higher loads because of the force of gravity as they act as a sort of hook. This holds with our database (Fig. 6), as the average upper and lower canines for hunting durophages are similar in size (upper canines are 0.5% larger), whereas scavenging durophages exhibit lower canines 4.1% larger than upper canines. The difference between these percentages was statistically significant (Mann–Whitney U test: *z* = 2.087; *p* = 0.037). Consequently, a tentative scenario to explain increased canines in scavenging durophages would be that natural selection adapted them for bone transport to cubs.

### Relationships between the carnassial length and the canine size in living hyenas

An essential point in the present discussion is that food transport to dens and social killing is related to living hyenas, which can be considered as the functional link between carnassial length and canine size observed in the present study. In fact, after a period of 11 days in birth dens, spotted hyena cubs are transferred to the communal den of the clan, where they are suckled by their respective mothers until the end of denning period (East, Hofer & Turk, 1989), which takes place when cubs are 12–15 months (Kruuk, 1972; Mills, 1990). Either because there is very little food to carry back after the scramble competition at ungulates kill sites, or because the transported food for cubs can be stolen by higher-ranking conspecifics (Holekamp & Smale, 1990), provisioning cubs is penalized by natural selection, whereas suckling is favored in spotted hyenas. In other words, gregariousness increases kill success, but it leads to cubs having to be fed only milk. This strategy is in agreement with elongated carnassials for a given canine size. On the contrary, brown hyenas being solitary feeders can supplement the diet of their cubs, and allosuckling has been observed (Mills, 1990). Consequently, the strategy of scavengers would be the opposite: to increase canines for a given carnassial length to adapt them to transport food to cubs. Because the correlation between carnassials and canines is reflected in the third principal component (including extant and extinct durophages), it is not unreasonable that these ethological features were also present in the extinct species.

### Evidence from extinct hyenas

In accordance with our findings, taphonomic, paleoecologic, and ecomorphologic studies on *Pachycrocuta brevirostis* in Venta Micena (Early Pleistocene from Spain) indicate that it was a solitary scavenger well adapted to dismembering and transporting large pieces of ungulate carcasses without dragging to their denning sites to be fractured (Palmqvist & Arribas, 2001; Palmqvist et al., 2011). Fossil assemblages accumulated by the giant hyena *P. brevirostris* are also congruent with the expectations from its large canines according to the transport hypothesis. Their earthen ground dens showed thousands of bones accumulated over short periods (Arribas & Palmqvist, 1998; Palmqvist & Arribas, 2001; Mazza, Bertini & Magi, 2004). The number of bones in earthen ground dens of *P. brevirostris* were equivalent to those observed for rock cave dens of brown and striped hyenas (mean values of 3659.3 bones per den) and were considerably higher than those for brown hyena earthen ground dens (mean values of 248.8 bones per den) according to the database compiled by Lansing et al. (2009). Bone density in *P. brevirostris* open-air accumulations could reach from 90 to 500 bones per m^2^ (Arribas & Palmqvist, 1998; Mazza, Bertini & Magi, 2004). Allochthonous species have also been found in their dens (Arribas & Palmqvist, 1998; Palmqvist et al., 2008), which indicated that *P. brevirostris* also prospected large areas.

On the other hand, it has been argued that *C. crocuta spelaea* was also a frequent bone collector (e.g., Diedrich & Zák, 2006); however, in the present analysis it has been classified into the group of hunting durophages. It is important to note here that the vast majority of the accumulations assigned to this hyena are in caves, which could be the result of a thousand years of either continuous occupation or cycles of abandonment and re-occupation. Additionally, bone preservation is better in caves (Lansing et al., 2009). Nevertheless, figures of only a few hundred bones are quite common in cave rock assemblages interpreted as *C. crocuta spelaea* dens. For example, according to Diedrich (2011), Teufelskammer Cave, Hohle Stein Cave, and Wilhelms Cave in Germany contained 199, 151, and 321 identified specimens, respectively. A *C. crocuta spelaea* open-air den described by Diedrich (2006) (and therefore comparable to the dens of *P. brevirostris* mentioned above) had only 152 bones lying on several sedimentary depression structures along a 40 m long outcrop. In conclusion, taphonomic analyses are congruent with the ecomorphological inferences obtained here.

An interesting question that arises from this study is how the relationship between carnassials and canines was shaped over the course of evolution. Given that *A. eximia* has carnassials similar to living hunting durophages, it is not unreasonable that this species was an incipient social hunter; however, its generalized canines indicate a certain ability to transport carcass remains to cubs. Mills (1990) and Holekamp & Smale (1990) consider extensive provisioning as the ancestral condition in Hyaenidae, which would be congruent with this result for *A. eximia*. In any case, this species cannot be considered as being ecologically equivalent to the modern *C. crocuta*. The first true representative of this ecotype is *C. dietrichi* from the Early Pliocene of East Africa (Petter & Howell, 1989). *C. eturono*, described by Werdelin & Lewis (2008), partially overlaps its stratigraphic range with *C. dietrichi* (Werdelin & Lewis, 2005). Nevertheless, *C. eturono* post-canine dentition indicates high specialization as a hunting durophagous species, even more than living *C. crotuta* (Coca-Ortega & Pérez-Claros, 2019). Canines of *C. eturono* are unknown, and consequently, this species was not analyzed here; however, according to its post-canine dentition, their canines should be relatively small.

The relationship between carnassials and canines observed here for the living scavenging durophages is exhibited by *P. perrieri*, a widely spread taxon across Europe, Asia, and North Africa, which is known from the beginning of the Pliocene (Werdelin & Solounias, 1991). Perhaps, *Allohyaena kadici*, a Tortonian giant hyena from Central Europe could have presented the same adaptation as *P. perrieri*. The post-canine dentition of *A. kadici* indicates that it was a scavenger (Coca-Ortega & Pérez-Claros, 2019); however, it has not been included here because there are no published data regarding its canines. Consequently, both hunting and scavenging adaptations were present at least from the Zanclean. Perhaps it is not mere coincidence that these niches appear just at such age. In fact, at the end of the Miocene and the beginning of the Pliocene, this family underwent a profound reconfiguration as many of the ecotypes experienced a drastic drop in diversity, and many of them disappeared (Werdelin & Turner, 1996; Werdelin & Solounias, 1996), which was certainly associated with relevant ecological changes (Agustí & Antón, 2002).

## Conclusions

Living durophagous hyenas are representatives of two different adaptive trends closely related to sociality, which can be evidenced by analyzing the dentition of this family as a whole. On the one hand, social hunting durophagous hyaenids such as living *C. crocuta*, have elongated carnassials as a consequence of scramble competition at feeding and reduced sizes of canines. On the other hand, scavenging durophagous species such as living striped and brown hyenas, are solitary foragers with reduced carnassials and enlarged canines, particularly the lower ones. As a tentative hypothesis, this latter feature is considered an adaptation to carry food to dens because as solitary animals, there is no risk of food being stolen by conspecifics. Under this scenario, the lower canines would be used as hooks.

Both sets of adaptations were delayed in the upper dentition with respect to the lower teeth, which could indicate a higher degree of constraint on the former, resulting in a mosaic evolution. These two trends exhibited by fully developed bone cracker hyenas can be traced to the Early Pliocene.

##  Supplemental Information

10.7717/peerj.10541/supp-1 Table S1 Individual measurements (mm) of the upper and lower canines for the species analyzed in this study The references regarding their proveniences are in Coca-Ortega & Pérez-Claros (2019). If the original source only provides a mean, its corresponding sample size is in brackets.

10.7717/peerj.10541/supp-2 Table S2 Means for the variables analyzed for living canids and felids, as well as the durophagous hyaenids, studied The numbers in brackets represent sample sizes. Asterisks in body mass (BM) indicate estimated values.

## Additional Information and Declarations

### Data Availability

The following information was supplied regarding data availability: All the data are available in tables and in the Supplemental Files.

## References

[ref-1] Agustí J, Antón M (2002). Mammoths, sabertooths, and hominids: 65 million years of mammalian evolution in Europe.

[ref-2] Alam MS, Khan JA (2015). Food habits of Striped Hyena (Hyaena hyaena) in a semi-arid conservation area of India. Journal of Arid Land.

[ref-3] Antón M (2013). Sabertooth.

[ref-4] Arribas A, Palmqvist P (1998). Taphonomy and palaeoecology of an assemblage of large mammals: hyaenid activity in the lower Pleistocene site at Venta Micena (Orce, Guadix-Baza Basin, Granada, Spain). Geobios.

[ref-5] Boydston EE, Morelli TL, Holekamp KE (2001). Sex differences in territorial behavior exhibited by the spotted hyena (Hyaenidae, Crocuta crocuta). Ethology.

[ref-6] Christiansen P (2007). Canine morphology in the larger Felidae: implications for feeding ecology. Biological Journal of the Linnean Society.

[ref-7] Christiansen P (2008). Feeding ecology and morphology of the upper canines in bears (Carnivora: Ursidae). Journal of Morphology.

[ref-8] Christiansen P, Adolfssen JS (2005). Bite forces, canine strength and skull allometry in carnivores (Mammalia, Carnivora). Journal of Zoology.

[ref-9] Christiansen P, Wroe S (2007). Bite forces and evolutionary adaptations to feeding ecology in carnivores. Ecology.

[ref-10] Coca-Ortega C, Pérez-Claros JA (2019). Characterizing ecomorphological patterns in hyenids: a multivariate approach using postcanine dentition. PeerJ.

[ref-11] Cooper SM, Holekamp KE, Smale L (1999). A seasonal feast: long-term analysis of feeding behaviour in the spotted hyaena (*Crocuta crocuta*). African Journal of Ecology.

[ref-12] Diedrich CG (2006). The *Crocuta crocuta spelaea* (Goldfuss 1823) population from the early Late Pleistocene hyena open air prey deposit site Biedensteg (Bad Wildungen, Hess, NW Germany), a contribution to their phylogenetic position, coprolites and prey. Cranium.

[ref-13] Diedrich CG (2011). The *Crocuta crocuta spelaea* (Goldfuss 1823) population and its prey from the Late Pleistocene Teufelskammer Cave hyena den besides the famous Paleolithic Neandertal Cave (NRW, NW Germany). Historical Biology.

[ref-14] Diedrich CG, Zák K (2006). Prey deposits and den sites of the Upper Pleistocene hyena *Crocuta crocuta spelaea* (Goldfuss, 1823) in horizontal and vertical caves of the Bohemian Karst (Czech Republic). Bulletin of Geosciences.

[ref-15] East M, Hofer H, Turk A (1989). Functions of birth dens in spotted hyaenas (*Crocuta crocuta*). Journal of Zoology.

[ref-16] Ewer RF (1954). XXXI.—some adaptive features in the dentition of hyaenas. Annals and Magazine of Natural History.

[ref-17] Faure JPB, Holmes NJ, Watson LH, Hill RA (2019). Brown Hyaena (*Parahyaena brunnea*) diet Composition from Zingela Game Reserve, Limpopo Province, South Africa. African Zoology.

[ref-18] Figueirido B, MacLeod N, Krieger J, De Renzi M, Pérez-Claros JA, Palmqvist P (2011). Constraint and adaptation in the evolution of carnivoran skull shape. Paleobiology.

[ref-19] Figueirido B, Tseng ZJ, Martín-Serra P (2013). Skull shape evolution in durophagous carnivorans. Evolution.

[ref-20] Gould SJ, Vrba ES (1982). Exaptation—a missing term in the science of form. Paleobiology.

[ref-21] Hammer Ø, Harper DAT, Ryan PD (2001). PAST: Paleontological statistics software package for education and data analysis. Palaeontologia Electronica.

[ref-22] Henschel JR, Skinner JD (1991). Territorial behaviour by a clan of spotted hyaenas *Crocuta crocuta*. Ethology.

[ref-23] Holekamp KE, Smale L (1990). Provisioning and food sharing by lactating spotted hyenas, Crocuta crocuta (Mammalia: Hyaenidae). Ethology.

[ref-24] Holekamp KE, Smale L, Berg R, Cooper SM (1997). Hunting rates and hunting success in the spotted hyena (*Crocuta crocuta*). Journal of Zoology.

[ref-25] Jolliffe IT (1982). A note on the use of principal components in regression. Journal of the Royal Statistical Society: Series C (Applied Statistics).

[ref-26] Jolliffe IT (2002). Principal component analysis.

[ref-27] Kerbis-Peterhans JC, Horwitz LK (1992). A bone assemblage from a striped hyaena (*Hyaena hyaena*) den in the Negev Desert, Israel. Israel Journal of Ecology and Evolution.

[ref-28] Kruuk H (1972). The Spotted Hyena: a study of predation and social behavior.

[ref-29] Kruuk H (1976). Feeding and social behaviour of the striped hyaena (*Hyaena vulgaris* Desmarest). African Journal of Ecology.

[ref-30] Kuhn B (2005). The faunal assemblages and taphonomic signatures of five striped hyaena (*Hyaena hyaena syriaca*) dens in the desert of eastern Jordan. Levant.

[ref-31] Kuhn BF, Wiesel I, Skinner JD (2008). Diet of brown hyaenas (*Parahyaena brunnea*) on the Namibian coast. Transactions of the Royal Society of South Africa.

[ref-32] Lansing SW, Cooper SM, Boydston EE, Holekamp KE (2009). Taphonomic and zooarchaeological implications of spotted hyena (*Crocuta crocuta*) bone accumulations in Kenya: a modern behavioral ecological approach. Paleobiology.

[ref-33] Leakey LN, Milledge SAH, Leakey SM, Edung J, Haynes P, Kiptoo DK, McGeorge A (1999). Diet of striped hyaena in northern Kenya. African Journal of Ecology.

[ref-34] Macdonald DW (1978). Observations on the behaviour and ecology of the striped hyaena, Hyaena hyaena, in Israel. Israel Journal of Ecology and Evolution.

[ref-35] Maude G, Mills MGL (2005). The comparative feeding ecology of the brown hyaena in a cattle area and a national park in Botswana. South African Journal of Wildlife Research.

[ref-36] Mazza PP, Bertini A, Magi M (2004). The late Pliocene site of Poggio Rosso (central Italy): taphonomy and paleoenvironment. Palaios.

[ref-37] Mills MGL (1983). Mating and denning behaviour of the brown hyaena Hyaena brunnea and comparisons with other Hyaenidae. Zeitschrift für Tierpsychologie.

[ref-38] Mills ML (1984). The comparative behavioural ecology of the brown hyaena *Hyaena brunnea* and the spotted hyaena *Crocuta crocuta* in the southern Kalahari. Koedoe.

[ref-39] Mills MGL (1990). Kalahari hyaenas: the behavioural ecology of two species.

[ref-40] Mills MGL, McCullough DR, Barrett RH (1992). A comparison of methods used to study food habits of large African carnivores. Wildlife 2001: populations.

[ref-41] Mills MGL, Mills ME (1978). The diet of the brown hyaena *Hyaena brunnea* in the southern Kalahari. Koedoe.

[ref-42] Mondal PCK, Sankar K, Qureshi Q (2012). Food habits of golden jackal (*Canis aureus*) and striped hyena (*Hyaena hyaena*) in Sariska Tiger Reserve, Western India. World Journal of Zoology.

[ref-43] Owens MJ, Owens DD (1978). Feeding ecology and its influence on social organization in brown hyenas (*Hyaena brunnea*, Thunberg) of the central Kalahari Desert. African Journal of Ecology.

[ref-44] Palmqvist P, Arribas A (2001). Taphonomic decoding of the paleobiological information locked in a lower Pleistocene assemblage of large mammals. Paleobiology.

[ref-45] Palmqvist P, Martínez-Navarro B, Pérez-Claros JA, Torregrosa V, Figueirido B, Jiménez-Arenas JM, Espigares MP, Ros-Montoya S, De Renzi M (2011). The giant hyena *Pachycrocuta brevirostris*: modelling the bone-cracking behavior of an extinct carnivore. Quaternary International.

[ref-46] Palmqvist P, Pérez-Claros JA, Janis CM, Figueirido B, Torregrosa V, Gröcke DR (2008). Biogeochemical and ecomorphological inferences on prey selection and resource partitioning among mammalian carnivores in an early Pleistocene community. Palaios.

[ref-47] Petter G, Howell FC (1989). Une nouvelle espèce du genre Crocuta Kaup (Mammalia, Carnivora, Hyaenidae) dans la faune pliocène de Laetoli (Tanzanie): *Crocuta dietrichi* n. sp.; origine du genre. Comptes rendus de l’Académie des sciences. Série 2. Mécanique, Physique, Chimie, Sciences de l’univers, Sciences de la Terre.

[ref-48] Pokines JT, Kerbis-Peterhans JCK (2007). Spotted hyena (*Crocuta crocuta*) den use and taphonomy in the Masai Mara National Reserve, Kenya. Journal of Archaeological Science.

[ref-49] Seilacher A (1970). Arbeitskonzept zur konstruktions-morphologie. Lethaia.

[ref-50] Skinner JD (2006). Bone collecting by hyaenas: a review. Transactions of the Royal Society of South Africa.

[ref-51] Skinner JD, Haupt MA, Hoffmann M, Dott HM (1998). Bone collecting by brown hyaenas *Hyaena brunnea* in the Namib desert: rate of accumulation. Journal of Archaeological Science.

[ref-52] Skinner JD, Henschel JR, Van Jaarsveld AS (1986). Bone-collecting habits of spotted hyaenas *Crocuta crocuta* in the Kruger National Park. African Zoology.

[ref-53] Skinner JD, Ilani G (1979). The striped hyaena *Hyaena hyaena* of the Judean and Negev deserts and a comparison with the brown hyaena *H. brunnea*. Israel Journal of Zoology.

[ref-54] Skinner JD, Van Aarde RJ (1991). Bone collecting by brown hyaenas *Hyaena brunnea* in the central Namib Desert, Namibia. Journal of Archaeological Science.

[ref-55] Smith JE, Kolowski JM, Graham KE, Dawes SE, Holekamp KE (2008). Social and ecological determinants of fission–fusion dynamics in the spotted hyaena. Animal Behaviour.

[ref-56] Turner A, Antón M, Werdelin L (2008). Taxonomy and evolutionary patterns in the fossil Hyaenidae of Europe. Geobios.

[ref-57] Van Valkenburgh B, Gittleman JL (1989). Carnivore dental adaptations and diet: a study of trophic diversity within guilds. Carnivore behavior, ecology, and evolution.

[ref-58] Van Valkenburgh B (1991). Iterative evolution of hypercarnivory in canids (Mammalia: Carnivora): evolutionary interactions among sympatric predators. Paleobiology.

[ref-59] Van Valkenburgh B (1996). Feeding behavior in free-ranging, large African carnivores, large African carnivores. Journal of Mammalogy.

[ref-60] Van Valkenburgh B (2009). Costs of carnivory: tooth fracture in Pleistocene and Recent carnivorans. Biological Journal of the Linnean Society.

[ref-61] Van Valkenburgh B, Ruff CB (1987). Canine tooth strength and killing behaviour in large carnivores. Journal of Zoology.

[ref-62] Wagner AP, Frank LG, Creel S (2008). Spatial grouping in behaviourally solitary striped hyaenas, Hyaena hyaena. Animal Behaviour.

[ref-63] Werdelin L, Lewis ME (2005). Plio-Pleistocene Carnivora of eastern Africa: species richness and turnover patterns. Zoological Journal of the Linnean Society.

[ref-64] Werdelin L, Lewis ME (2008). New species of Crocuta from the early Pliocene of Kenya, with an overview of early Pliocene hyenas of eastern Africa. Journal of Vertebrate Paleontology.

[ref-65] Werdelin L, Solounias N (1991). The Hyaenidae: taxonomy, systematics and evolution. Fossils and Strata.

[ref-66] Werdelin L, Solounias N, Bernor RL, Fahlbusch V, Rietschel S (1996). The evolutionary history of hyenas in Europe and western Asia during the Miocene. The evolution of Western Eurasian Neogene mammal faunas.

[ref-67] Werdelin L, Turner A (1996). Turnover in the guild of larger carnivores in Eurasia across the Miocene-Pliocene boundary. Acta Zoologica Cracoviensia.

[ref-68] Wroe S, McHenry C, Thomason J (2005). Bite club: comparative bite force in big biting mammals and the prediction of predatory behaviour in fossil taxa. Proceedings of the Royal Society B: Biological Sciences.

